# Siglecs Facilitate HIV-1 Infection of Macrophages through Adhesion with Viral Sialic Acids

**DOI:** 10.1371/journal.pone.0024559

**Published:** 2011-09-08

**Authors:** Zhongcheng Zou, Ashley Chastain, Susan Moir, Jennifer Ford, Kathryn Trandem, Elena Martinelli, Claudia Cicala, Paul Crocker, James Arthos, Peter D. Sun

**Affiliations:** 1 Laboratory of Immunogenetics, National Institute of Allergy and Infectious Diseases, National Institutes of Health, Rockville, Maryland, United States of America; 2 Laboratory of Immunoregulation, National Institute of Allergy and Infectious Diseases, National Institutes of Health, Bethesda, Maryland, United States of America; 3 Cell Biology and Immunology, College of Life Sciences, University of Dundee, Dundee, United Kingdom; University Hospital Zurich, Switzerland

## Abstract

**Background:**

Human immunodeficiency virus type 1 (HIV-1) infects macrophages effectively, despite relatively low levels of cell surface-expressed CD4. Although HIV-1 infections are defined by viral tropisms according to chemokine receptor usage (R5 and X4), variations in infection are common within both R5- and X4-tropic viruses, indicating additional factors may contribute to viral tropism.

**Methodology and Principal Findings:**

Using both solution and cell surface binding experiments, we showed that R5- and X4-tropic HIV-1 gp120 proteins recognized a family of I-type lectin receptors, the **S**ialic acid-binding **i**mmuno**g**lobulin-like **lec**tins (Siglec). The recognition was through envelope-associated sialic acids that promoted viral adhesion to macrophages. The sialic acid-mediated viral-host interaction facilitated both R5-tropic pseudovirus and HIV-1_BaL_ infection of macrophages. The high affinity Siglec-1 contributed the most to HIV-1 infection and the variation in Siglec-1 expression on primary macrophages from different donors was associated statistically with sialic acid-facilitated viral infection. Furthermore, envelope-associated sialoglycan variations on various strains of R5-tropic viruses also affected infection.

**Conclusions and Significance of the Findings:**

Our study showed that sialic acids on the viral envelope facilitated HIV-1 infection of macrophages through interacting with Siglec receptors, and the expression of Siglec-1 correlated with viral sialic acid-mediated host attachment. This glycan-mediated viral adhesion underscores the importance of viral sialic acids in HIV infection and pathogenesis, and suggests a novel class of antiviral compounds targeting Siglec receptors.

## Introduction

Human immunodeficiency virus type 1 (HIV-1) infection of macrophages and T cells requires both CD4 and chemokine receptors [1]. While binding to CD4 provides mainly viral attachment to host cells, the interaction of the viral envelope protein gp120 with chemokine receptors initiates conformational changes leading to the fusion and entry of the virus [2]. Although CD4^+^ T cells are the major target of HIV, macrophages represent a potentially long-lived viral reservoir that may help the virus resist eradication [3], [4]. Furthermore, infected macrophages appear to contribute to dementia and neural dysfunction in infected individuals [5]. However, unlike CD4^+^ T cells, macrophages express relatively low levels of CD4 thereby complicating efficient viral entry [6]. Additional attachment factors, including carbohydrate receptors, have been proposed to facilitate infection of macrophages [7].

On their surface, macrophages express a number of C-type lectins, including the macrophage mannose receptor (MMR) and DEC-205. Although both the MMR and DEC-205 can capture HIV-1 through recognition of mannose-associated glycans on the viral envelope protein, binding to these receptors results primarily in non-productive infection or internalization of the virus for antigen presentation [8], [9], [10], [11]. In addition to the C-type lectins, macrophages also express a number of **S**ialic acid-binding **i**mmuno**g**lobulin-like **lec**tins (Siglec). In humans, the Siglec family is comprised of a total of 14 different genes that specifically recognize terminal sialic acids associated with both N- and O-linked glycosylations. They are expressed mainly on lymphoid and myeloid lineage cells where they promote cell-to-cell adhesion through binding to cell surface-associated sialic acids [12]. Siglecs are also known to recognize heavily glycosylated mucins and mucin-like domains [13], [14].

Like many enveloped retroviruses, the envelope protein of HIV-1 is heavily glycosylated. The crystal structure of the viral envelope protein revealed the molecular details of the CD4 binding site on gp120 [15], [16]. Mutations in gp120 that remove N-linked glycan sites on both HIV and SIV have resulted in viruses that are more sensitive to neutralization, suggesting a role of the viral glycan in shielding against host immune detection [17], [18], [19]. However, some of the mutants, especially those with multiple glycan mutations, also displayed much lower viral infectivity and syncytia-forming ability compared to the wild-type viruses, indicating a potential involvement of these glycans in viral entry.

The best studied example involving sialic acid in viral entry is the *Orthomyxoviridae* family of influenza viruses, which use hemagglutinin as the attachment receptor to bind to cell surface sialic acids [20], [21]. HIV and many other enveloped viruses do not encode hemagglutinin for sialic acid binding. Rather, these viruses can have N-terminal sialic acid bound to envelope-associated proteins, like gp120 on HIV-1. Interestingly, it has been shown that removal of cell surface sialic acids via neuraminidase treatment enhances HIV infection [22]. Recently, evidence suggests that sialoadhesin (Siglec-1) on a transfected monocytic cell line can interact with HIV-1 [23]. In addition, Siglec-1 expression is up-regulated on certain populations of monocytes upon HIV-1 infection [24]. It is not clear, however, whether Siglec-1 recognizes heavily glycosylated viral envelope proteins, such as HIV-1 gp120, and if the increased expression of Siglec-1 would facilitate viral attachment and entry. Here, we systematically evaluated the interactions between Siglecs and gp120 in solution, on receptor-transfected cells, and on primary monocyte-derived macrophages (MDM), and examined the potential importance of Siglec-gp120 interactions in HIV-1 infection.

## Results

### Recombinant Siglec receptors bound soluble gp120 from both R5- and X4-tropic strains of HIV-1 in solution

**Figure 1:**
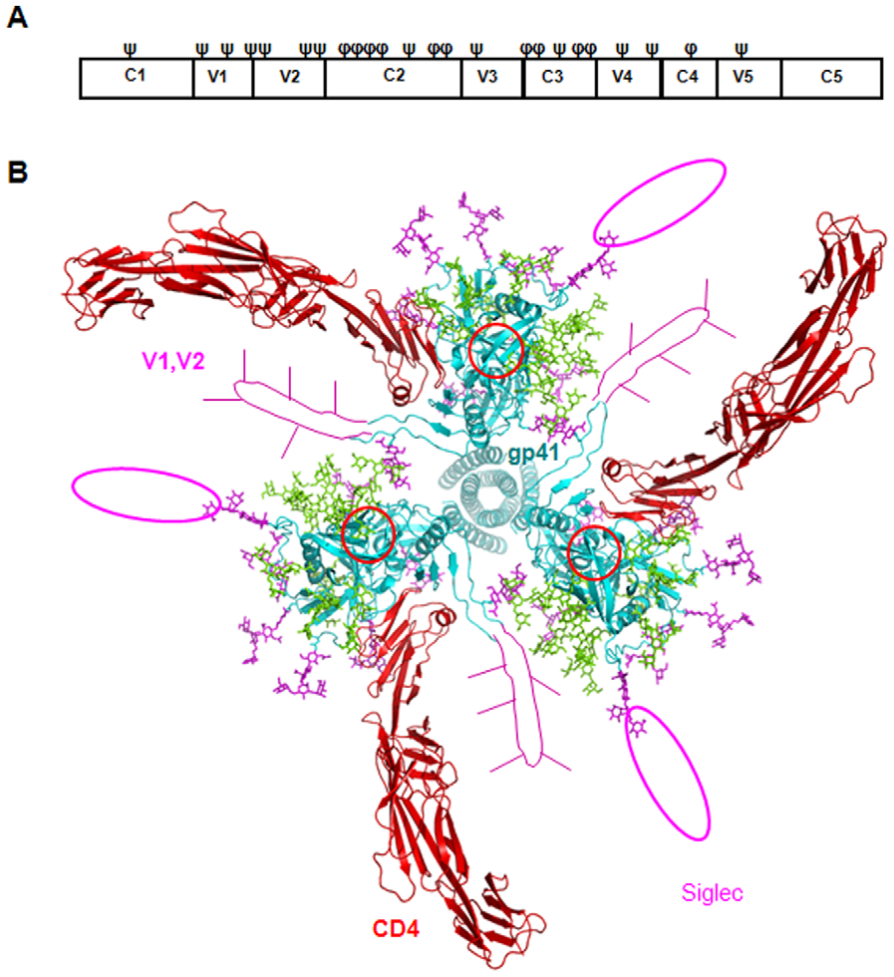
A structural model of gp120 and associated glycans. (A) Location of glycosylation sites on the IIIB strain of HIV-1 gp120 with complex (ψ) and high mannose or hybrid (ϕ) types. (B) Distribution of glycans on HIV-1 gp120 and gp41 trimer (cyan) in complex with CD4 (red). Coordinates for the gp120 trimer model with two-domain CD4 was kindly provided by P. Kwong [27]. The four-domain CD4 model (pdb code 1WIO) is placed in the complex by superposition of the two N-terminal domains. The view is from host cell down to virus. The V1 and V2 loops are drawn in the figure, as both loops are missing in the crystal structure of the gp120 and CD4 complex (pdb code 1GC1). Subunits are color-coded cyan for gp41 and gp120, red for CD4, magenta for complex-type carbohydrates, and green for high mannose and hybrid type-carbohydrates. The V3 loop region is denoted by a red circle.

gp120, a surface envelope protein on HIV-1, encodes over 20 N-linked glycosylation sites. Many of these sites are conserved between CCR5- (R5) and CXCR4-tropic (X4) strains of the virus [25], [26]. Approximately half of these sites carry a complex type of carbohydrate with terminal sialic acid modifications [25]. The others are a mixture of high mannose or hybrid types of carbohydrates. Interestingly, the glycans on the five gp120 variable loops are almost exclusively of the complex type (Figure 1A). When the glycans are displayed onto a model of a gp120 trimer [27], the complex types are distributed evenly along the outer rim of the trimer, and are potentially available to bind sialic acid receptors (Figure 1B). This raises an intriguing possibility that sialic acids on gp120 may facilitate viral attachment and entry through direct binding to Siglecs.

**Figure 2:**
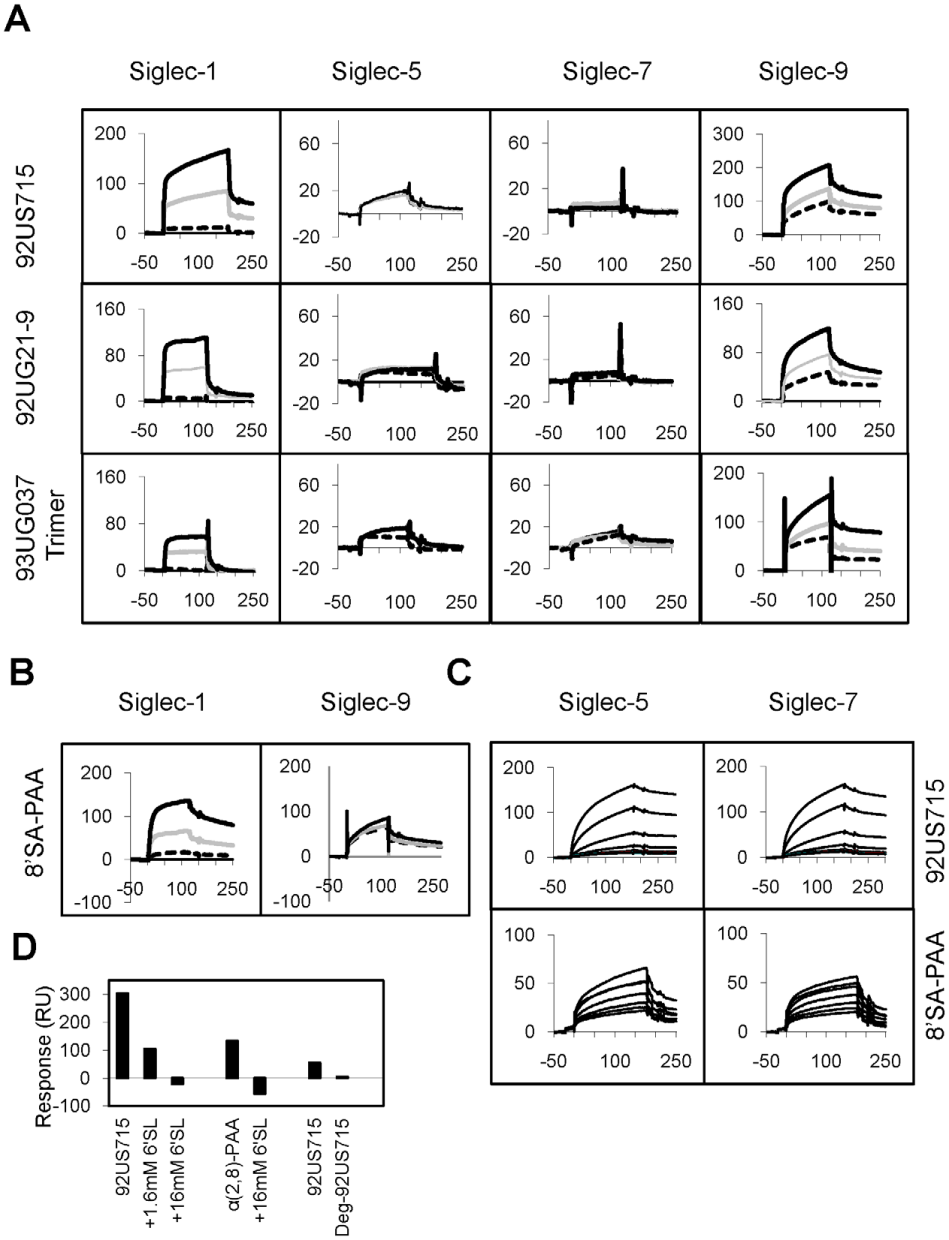
SPR experiments between gp120 and Siglecs. (A) Binding of recombinant gp120 from 92US715 (0.65 µM), 92UG21-9 (0.49 µM), and 93UG037 (0.2 µM) HIV-1 isolates onto protein A-captured recombinant Siglec-1, -5, -7, and -9 Fc fusion proteins. The sensorgrams in each panel represent individual Siglecs captured in the order of decreasing surface densities between black, grey and dashed curves. Flow cell 1 has immobilized protein A but no captured receptor (blank). The binding curves of gp120 to captured Siglec-3 are shown in Figure S2. (B) Binding of 10 µM α(2,8)-sialyllactose-PAA (8′SA-PAA) onto protein A-captured recombinant Siglec-1 and -9 Fc fusion proteins in similar settings as (A). (C) Binding of serial dilutions of 92US715 gp120 between 0.43–0.054 µM or 8′SA-PAA between 0.062–20 µM onto immobilized Siglec-5 and -7 under high immobilization density. (D) Binding of 92US715 gp120 and 8′SA-PAA to protein A-captured Siglec-9 in the presence of α(2,6)-sialyllactose (6′-SL), and the effect of deglycosylation of 92US715 gp120 on the binding of protein A-captured Siglec-9. All sensorgrams are shown in response units (vertical axis) versus sample injection time (horizontal axis) in seconds. Dissociation constants are listed in Table 1.

Since Siglec receptors are known to recognize sialic acids on mucin-like glycoprotein, we used a surface plasmon resonance (SPR) technique to investigate if gp120-associated sialic acids can be recognized by Siglecs [28], [29]. We performed direct binding between various recombinant gp120 proteins and the five soluble human Siglec receptors (Siglec-1, -3, -5, -7, and -9) expressed on monocytes. When captured on an immobilized protein A sensorchip, Siglec-1 and -9 Fc fusion receptors bound readily to various gp120 envelope proteins with affinities varied between 0.01–1 µM (Figure 2A). Their gp120 binding affinities were similar to their binding affinities to polyacrylamide-conjugated sialoglycans (Table 1, Figure 2B), suggesting that the observed Siglec-gp120 interactions were specific. Interestingly, both Siglec receptors recognized multiple strains of gp120, albeit with different affinities, from both R5- and X4-tropic viruses. For example, Siglec-9 bound to the R5-tropic envelopes from 93UG037, SIVsmm PBj1.9, and 92US715 isolates with affinities between 0.02 and 0.3 µM while it bound to the X4-tropic envelopes from 92UG21-9 and NL4-3 strains of HIV-1 with 0.05 and 0.12 µM affinities, respectively.

**Table 1:** Dissociation constants (K_D_) for gp120 binding to Siglec Receptors.

K_D_ (µM) for HIV-1 strains									
	R5-Tropic				X4-Tropic		PAA^4^		
Isolates	UG037^1^	PBJ^1^	US715^1^	Trimer^2^	UG21^1^	NL4-3^1^	3′SL	6′SL	8′SA
Clades	A	SIV	B	A	A	B			
Siglecs									
Moderate Immobilization Density (2000–7000 RU)									
Siglec-1	6.3	0.12	0.13	0.9	0.08	0.033	0.5	2.0	2.3
Siglec-3	ND^3^	54.5	57.8	ND	3.8	ND	4.5	7.0	5.0
Siglec-5	20.3	50.5	9.4	5.1	47.1	ND	1.6	0.5	1.8
Siglec-7	9.9	35.4	53.4	2.7	49.2	ND	1.5	1.0	0.9
Siglec-9	0.10	0.27	0.023	0.08	0.05	0.012	0.5	0.3	2.9

High Immobilization Density (12000–20000 RU)									
	UG037^1^	PBJ^1^	US715^1^	MW959^1^	UG21^1^	NL4-3^1^	3′SL	6′SL	8′SA
Siglec-3	0.051	0.18	0.008	0.012	0.08	0.009	13	2.1	4.8
Siglec-5	0.054	0.014	0.02	0.015	0.05	0.008	13	11.1	2.2
Siglec-7	0.094	0.019	0.04	0.02	0.05	0.013	1.7	2.5	1.9

1 The strain abbreviations with Genbank accession numbers in parenthesis are: UG037 for 93UG037 (U51190), PBJ for SIVsmm PBj1.9 (M31325), US715 for 92US715 (U04919), UG21 for 92UG21-9 (U08804), NL4-3 (M38432), MW959 for 93MW959 (Clade C, U08453).

2 The trimeric gp120 is from the 93UG037 strain. The recombinant protein was expressed in CHO cells fused with a trimerization domain from T4 bacteriophage fibritin [51], [52].

3 ND: Not Done.

4 The symbols for polyacrylamide (PAA)-conjugated model carbohydrates are 3′SL, 6′SL, and 8′SA for α(2,3)-sialyllactose-PAA, α(2,6)-sialyllactose-PAA, and Neu5Acα(α2,8)-Neu5Acα(α2,8)Neu5Acα-sp-PAA, respectively.

Furthermore, as gp120 exists in a trimeric state on HIV-1, we also examined the binding affinities of Siglec-1, -5, -7 and -9 to a trimeric gp120 derived from the 93UG037 isolate, and observed a three to five-fold increase in affinities compared to the monomeric gp120 from the same strain, with the exception of Siglec-9, that showed similar affinity with both the monomeric and trimeric gp120 (Table 1). Compared to Siglec-1 and -9, Siglec-3, -5, and -7 proteins displayed low affinity binding to gp120 when the receptors were captured at similar levels. However, the gp120 binding affinities for Siglec-3, -5, and -7 increased between 100- to 1000-fold when the receptors were immobilized at three to five-fold higher surface densities (Table 1, Figure 2C), indicating an avidity contribution to the Siglec-gp120 recognition. In contrast, the affinity of Siglecs for polyacrylamide-conjugated α(2,3)-sialyllactose, α(2,6)-sialyllactose, and α(2,8)-disialic acid was consistently in the micromolar range, independent of receptor immobilization density (Table 1). This profound avidity effect of Siglecs binding to gp120, but not to PAA-conjugated carbohydrates, indicates that the level of Siglec expression on the surface of a cell may affect sialic acid-mediated viral attachment.

To investigate whether Siglec-gp120 interactions were indeed mediated by sialic acid, we examined the binding between 92US715 gp120 and Siglec-9 in the presence of a competitor, α(2,6)-sialyllactose (6′SL). The binding of Siglec-9 to both gp120 and a control sialic acid polymer, α(2,8)-disialic acid polyacrylamide conjugate, was inhibited by 16 mM α(2,6)-sialyllactose (Figure 2D). Consistently, treating R5-tropic gp120 (92US715) with peptidyl-N-glycanase-F to enzymatically remove N-linked glycans also reduced gp120 binding to Siglec-9. Overall, the solution binding results showed that Siglec receptors recognized various recombinant gp120 proteins with Siglec-1 and -9 displaying higher binding affinities than Siglec-3, -5, and -7. The Siglec binding affinities also varied among gp120 derived from different HIV and SIV isolates.

### Siglec-transfected CHO cells recognized recombinant HIV-1 gp120

**Figure 3:**
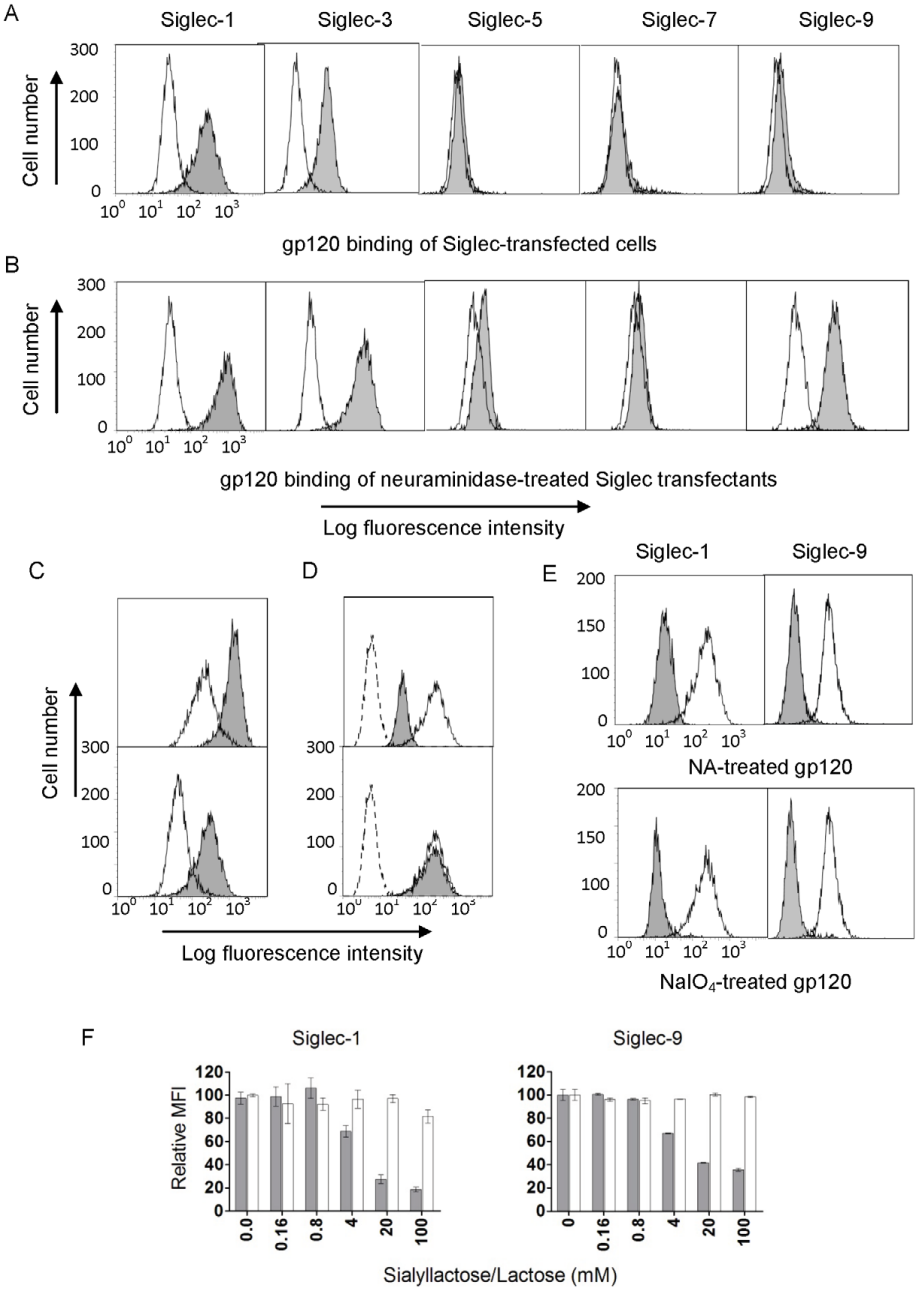
Recombinant biotin-labeled gp120 from the 93MW959 isolate (R5-tropic subtype C) binding to Siglec-transfected CHO cells. (A) Untransfected (open histogram) or Siglec-transfected CHO cells (grey histogram) were incubated with gp120 after being blocked with BSA and murine IgG, and then stained with PE-conjugated streptavidin. (B) The panels are similar to (A) except the cells were pretreated with neuraminidase (NA). (C) The Siglec-1 expression levels detected by an anti-Siglec-1 antibody (top panel) from clones 118 (open histogram) and 78 (grey histogram) of transfected CHO cells correlated with their binding to gp120 (bottom panel). (D) Binding of gp120 to Siglec-1 transfected CHO cells in the presence (grey histogram) or absence (open histogram) of 50 µg/mL human Siglec-1 blocking antibody (top) or pre-incubated with (grey histogram) or without (open histogram) 50 µg/mL recombinant soluble CD4 (bottom). Dotted lines correspond to staining with PE-conjugated streptavidin only. (E) Binding of gp120, untreated (open), NA or sodium periodate (NaIO_4_) treated (grey), to Siglec-1, or Siglec-9-transfected CHO cells. Siglec-9-transfected cells were pretreated with NA. (F) Binding of gp120 to Siglec-1 or Siglec-9 transfected CHO cells in the presence of titrating α2,3-/α2,6-sialyllactose (grey) or lactose (white).

To examine if the binding between Siglec receptors and gp120 observed in solution also occurred on the cell surface, we established stable CHO cell transfectants expressing human Siglec-1, -3, -5, -7 or -9 individually (Figure S1). Cell surface binding between a biotin-labeled recombinant soluble gp120 from the 93MW959 isolate (R5-tropic subtype C) and two of the Siglec-transfected cell lines (Siglec-1 and -3) was readily observed by FACS analysis (Figure 3A). Furthermore, when two CHO cell-transfectants with different Siglec-1 expression levels were compared, the fluorescence intensity of cell surface-bound gp120 correlated with the level of Siglec-1 expression (Figure 3C), and the gp120 binding was inhibited by a Siglec-1 blocking antibody (Figure 3D). This indicates that the recognition was indeed mediated by the transfected receptor. To address if the Siglec binding site on gp120 overlaps with that of CD4 binding, biotinylated gp120 was pre-incubated with a recombinant soluble CD4 prior to binding to Siglec-1 transfected CHO cells (Figure 3D). The result showed that the presence of CD4 did not affect the gp120 binding to Siglec-1, suggesting that Siglec-1 binding site on gp120 is separate from that of CD4. Neuraminidase (NA) treatment of Siglecs expressed on the cell surface often enhances their *trans*-ligand recognition, presumably due to the removal of *cis*-bound or “masking” sialic acids [30]. Siglec-1, which contains 17 extracellular domains, is the largest Siglec receptor and is likely least masked with sialic acids. Overall, Siglec-1, -3, -5 and -9 transfected CHO cells exhibited enhanced binding to recombinant 93MW959 gp120 when treated with neuraminidase (Figure 3B). In addition, when the recombinant gp120 was treated with neuraminidase to remove the envelope-associated sialic acids, binding to Siglec-1 and Siglec-9 transfectants was reduced significantly compared to untreated gp120 (Figure 3E). A similar reduction in binding was also observed when the soluble gp120 was treated by mild periodate oxidation, which truncates the glycerol side chain of sialic acids and leads to the loss of Siglec recognition [31], [32] (Figure 3E). To further demonstrate that Siglec binding to HIV-1 depends on gp120-associated sialic acids, CHO cell binding experiments were carried out in the presence of either sialyllactose, a known ligand of Siglec receptors [28], or lactose. Our results showed that sialyllactose inhibited the 93MW959 gp120 attachment to the Siglec-1 or -9 transfected cells while lactose, an analogue lacking sialic acid, did not affect the gp120 binding (Figure 3F).

### Siglec receptors on macrophages recognized soluble gp120

**Figure 4:**
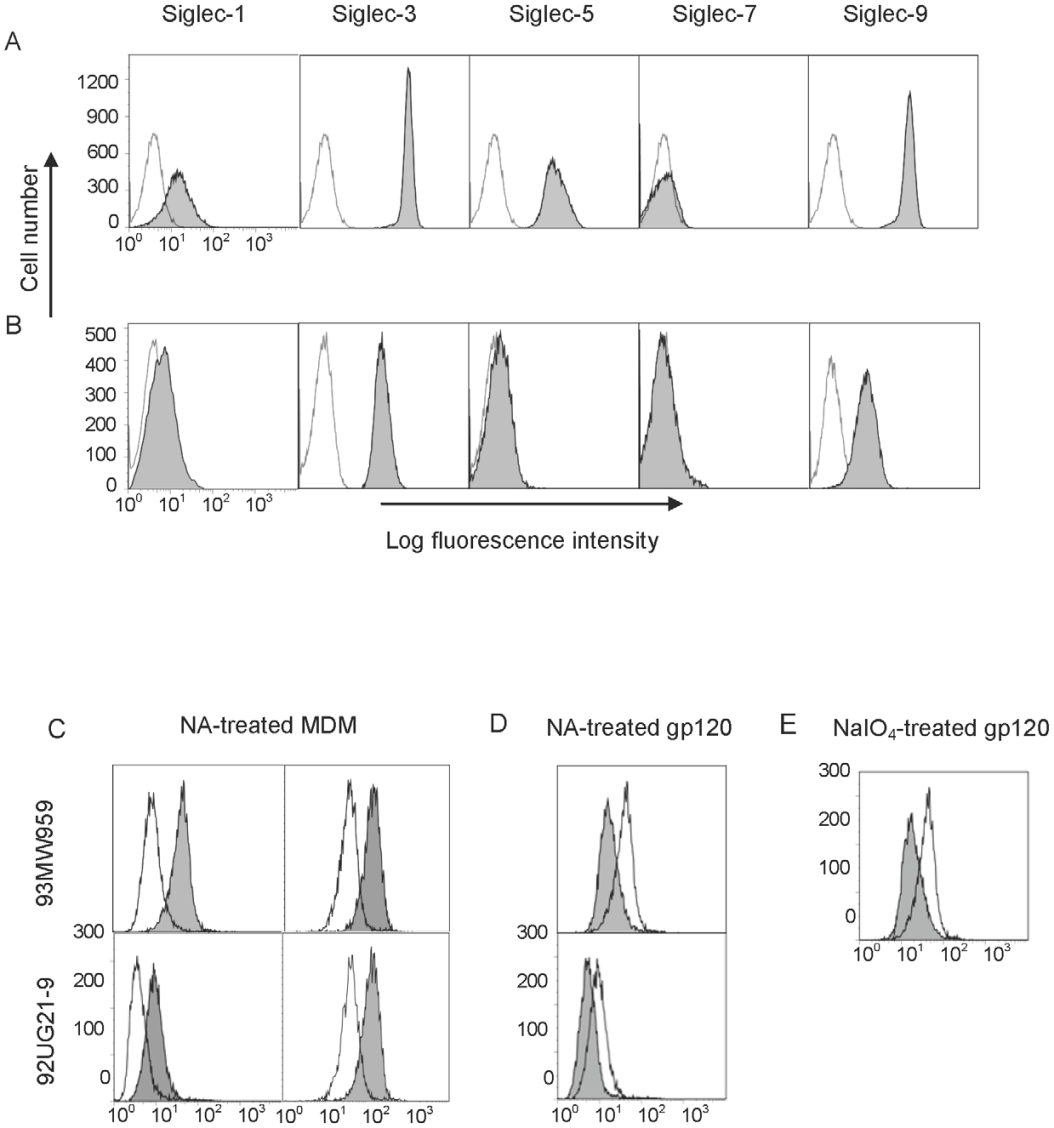
Siglec expression and recombinant gp120 binding to human monocytes or MDM. (A) The expression of Siglecs (grey) on CD14^+^ monocytes. (B) The expression of Siglecs (grey) on CD14^+^ MDM. The controls were stained with PE-conjugated isotype-matched IgGs. All samples were blocked with mouse IgG prior staining. (C) Binding of biotinylated gp120 from 93MW959 (top) or 92UG21-9 (bottom) isolates to NA-treated (grey) or untreated (open) MDM in the presence (left) or absence (right) of a CD4-blocking antibody (OKT4). (D, E) Binding of biotinylated 93MW959 (top) or 92UG21-9 (bottom) recombinant gp120, untreated (open histogram) or treated (grey histogram) with NA (D) or NaIO_4_ (E) to NA treated MDM in the presence of a CD4-blocking antibody (OKT4).

Freshly isolated human monocytes were differentiated into macrophages using M-CSF and GM-CSF. The differentiated macrophages expressed a significant amount of Siglec-3 and -9, low levels of Siglec-1 and very little Siglec-5 and -7, compared with monocytes which express higher levels of Siglec 5 (Figures 4A & B). Using biotinylated gp120 from either an R5- (93MW959) or an X4-tropic (92UG21-9) isolate of HIV-1, we examined gp120 binding to monocyte-derived macrophages (MDM) both in the presence and absence of a CD4-blocking antibody. The results showed that gp120 binding was enhanced significantly on cells treated with neuraminidase (Figure 4C), consistent with the enhanced adhesion by Siglec receptors observed upon neuraminidase treatment in the transfected cells. Alternatively, when the soluble R5- or X4-tropic gp120 was treated with neuraminidase or sodium periodate, its binding to MDM was significantly reduced (Figures 4D & E). These results showed sialic acid-mediated binding of HIV-1 envelope protein to macrophages and suggest a potential contribution of viral sialic acid in HIV-1 attachment to the cell surface.

### Viral sialic acids facilitated single round R5-pseudotyped HIV-1 virus infection of macrophages

**Figure 5:**
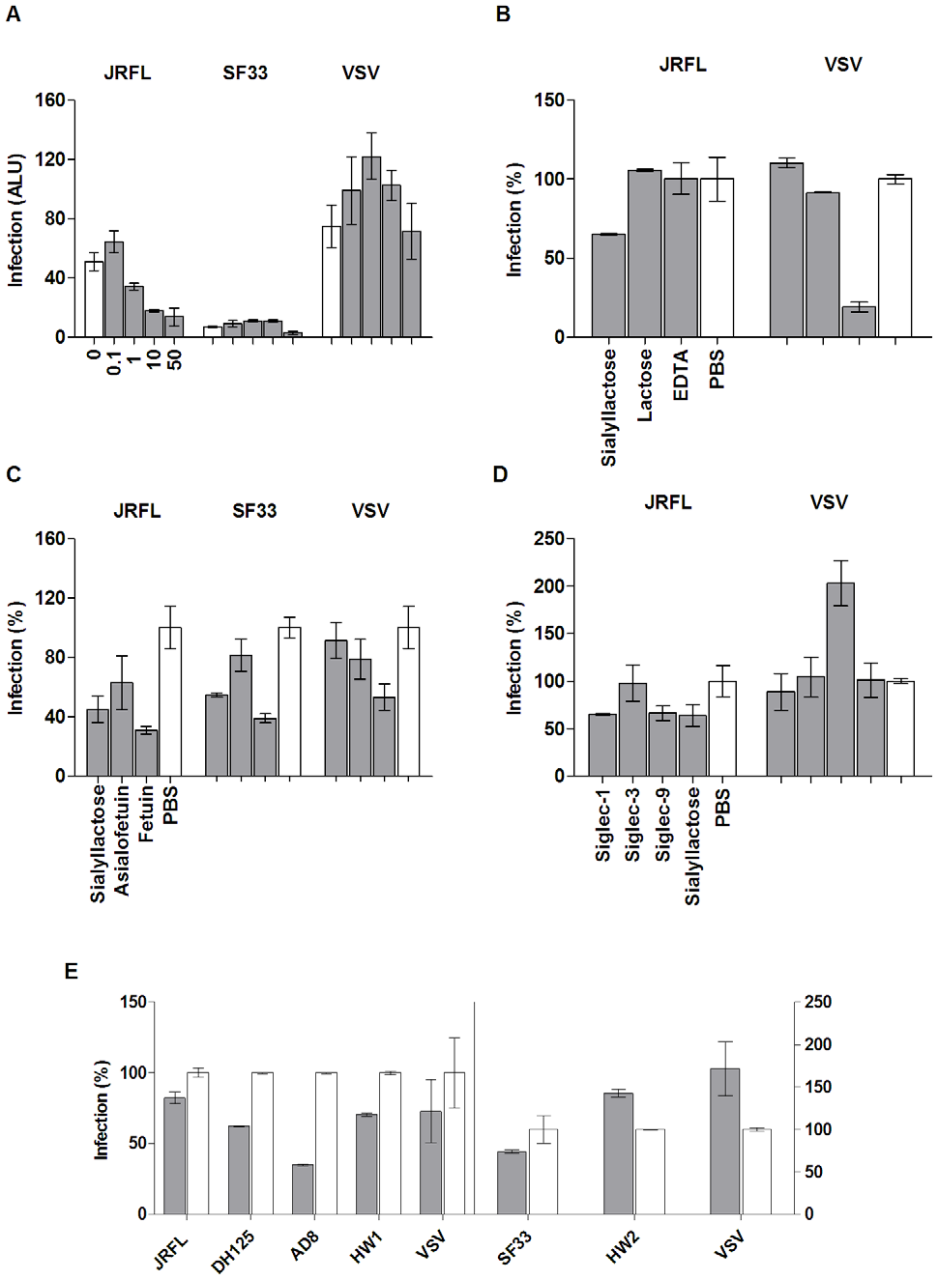
Single-round infection assays using R5- and X4-tropic HIV pseudoviruses. Except for panel A, which is shown in Arbitrary Light Units (ALU), the levels of viral entry (grey bars) are shown as percentage compared to their respective controls (open bars). (A) R5 and X4-tropic HIV and VSV entry into MDM in the presence of (from left to right) 0, 0.1, 1, 10, or 50 mg/mL sialyllactose (SL). (B) Entry of JRFL and VSV into MDM in the presence of 50 mg/mL SL or lactose, 1 mM EDTA, or PBS. (C) R5- and X4-tropic HIV-1 and VSV entry into MDM treated with 50 mg/mL SL, 1 mg/mL asialofetuin, 1 mg/mL fetuin, or PBS. (D) Entry of JRFL and VSV into MDM in the presence of 100 µg/mL recombinant Siglec-1, Siglec-3, Siglec-9, 50 mg/mL SL, or PBS. (E) Infection of MDM using R5-(left side) and X4-(right side) tropic HIV pseudoviruses treated with 1 mM NaIO_4_ (grey) or glycerol (white). Results are representative of one out of three experiments.

While recombinant HIV-1 gp120 from various strains bound to Siglec receptors in solution, on transfected cells, and on macrophages, it was not clear if the sialic acid-mediated gp120 binding would promote viral attachment and entry. To address this, we employed a luciferase-based single round infection assay using HIV-1 luciferase virus pseudotyped with envelopes derived from various HIV isolates or vesicular stomatitis virus (VSV) to infect MDM. Consistent with preferred tropism profiles and similar to previous observations [33], the infection of MDM by the R5-tropic JRFL pseudovirus routinely yielded 5–10 times higher luciferase activities (ALU) than that by a dose equivalent X4-tropic SF33 pseudovirus (Figure 5A). To examine the contribution of viral sialic acid to HIV-1 infection, we carried out the pseudovirus infection of MDM in the presence of sialyllactose or lactose, fetuin or its sialic acid-deprived form, asialofetuin. The addition of sialyllactose resulted in a dose-dependent inhibition of JRFL but not VSV pseudovirus infection (Figure 5A). Only the highest dose of sialyllactose inhibited the SF33-pseudotyped virus infection. Similar to the observed binding between a recombinant gp120 and Siglec receptors on transfected CHO cells, the pseudovirus infection was preferentially inhibited by 50 mg/ml sialyllactose not lactose and by 1 mg/ml fetuin not asialofetuin (Figure 5B & C). The failure of lactose and asialofetuin to inhibit the viral infection at equal concentrations compared to their sialo-analog demonstrated the importance of sialic acid in the infection.

Furthermore, the JRFL luciferase virus infection of MDM was partially inhibited by soluble recombinant Siglec-1,-3, -7, and -9 proteins (Figure 5D, Figure S3). The extent of inhibition by the individual Siglec proteins correlated with their binding affinities to sialic acid and gp120 (Table 1). Namely, the high affinity soluble Siglec-1 and -9 inhibited more than the low affinity Siglec-3. The broad involvement of viral sialic acids in HIV-1 infection is evident when MDM were infected with sodium periodate-treated pseudoviruses from multiple strains of HIV-1. Disrupting sialic acid with sodium periodate reduced the R5-pseudoviruses infection by 20–60% (Figure 5E). Since periodate-treated gp120 exhibited a reduced binding to MDM, the observed decrease in infection by periodate-treated virus is likely a result of reduced host attachment mediated by viral sialic acid.

### Siglec receptors contributed R5-pseudotyped HIV-1 virus infection of macrophages

**Figure 6:**
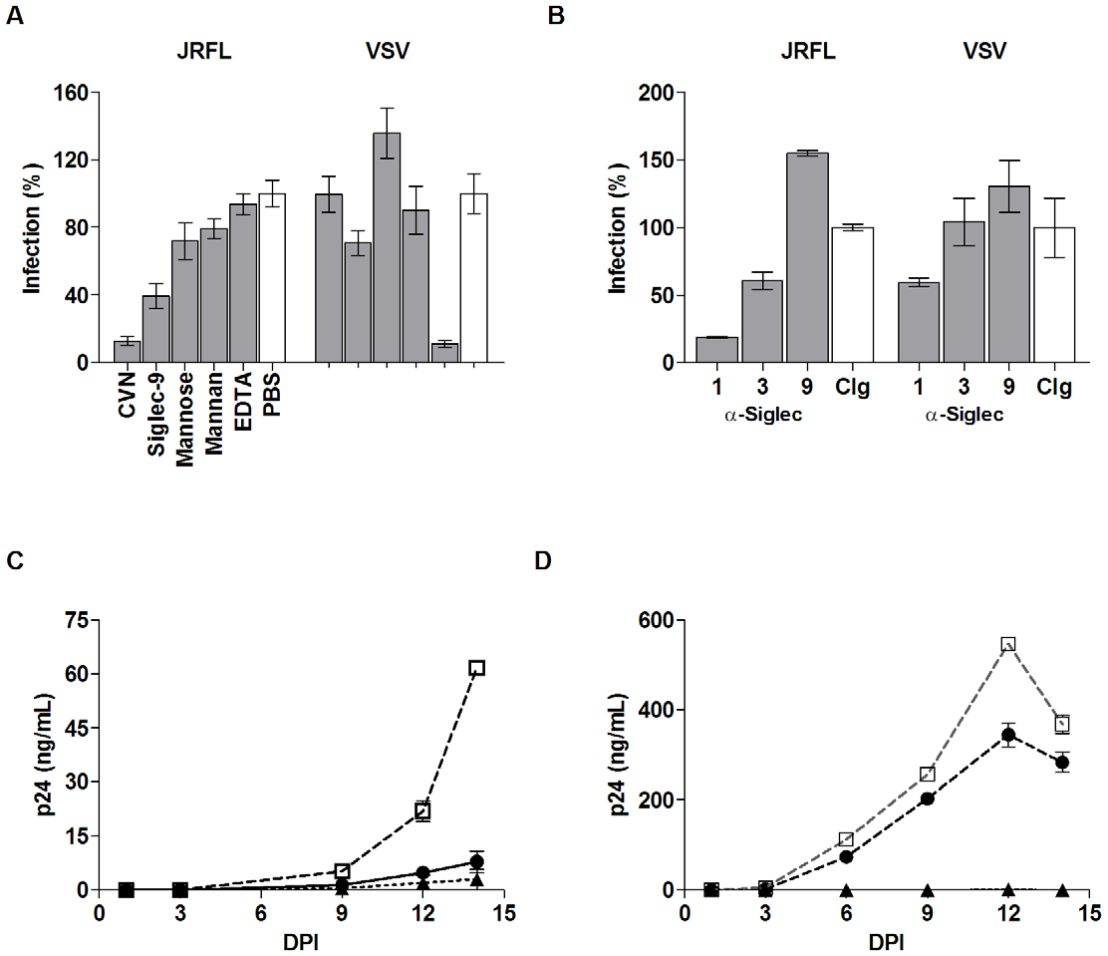
Effect of Siglec-specific compounds and blocking antibodies on pseudovirus and HIV-1_BaL_ infections of MDM. (A) Entry of JRFL and VSV into MDM in the presence of 500 ng/mL CVN, 100 µg/mL recombinant Siglec-9, 50 mg/mL mannose, 1 mg/mL mannan, 1 mM EDTA, or PBS. (B) Infection of MDM with JRFL and VSV pseudoviruses in the presence of 20 µg/mL Siglec-1 and -3, 100 µg/mL of Siglec-9 blocking antibodies, or 100 µg/mL control IgG (from mouse or sheep, open bars). (C & D) Infections of MDM with HIV-1_BaL_ (125 TCID_50_/10^6^ cells) were measured in p24 (ng/mL) at various days post-infection (DPI) in the presence of 50 mg/mL SL (black circles), 100 µg/mL T20 (black triangles), or PBS (open squares). Graphs A & B represent two infection experiments with different donor cells.

The involvement of carbohydrate recognition in HIV-1 infection has been controversial largely due to the use of cell lines in early research discoveries [34], [35]. This is also supported by the known antiviral effects of many carbohydrate binding agents (CBA), such as bacterial cyanovirin-N (CVN) and some plant lectins [36]. Consistent with previous publications, our infection experiment with JRFL pseudovirus was potently inhibited by CVN; VSV infection of MDM was not affected (Figure 6A). While CVN binding likely blocks viral access to both I-type and C-type lectin receptors on macrophages, we evaluated the potential contribution of C-type lectin receptors on MDM using mannose, mannan, and EDTA. Compared to the sialylated compounds, compounds specific for C-type lectin receptors exhibited less inhibition to JRFL-pseudovirus infection even though EDTA potently inhibited VSV pseudovirus infection (Figures 5B & 6A). This suggests a unique role for Siglecs recognition of viral sialylated glycans in HIV-1 attachment and entry.

To further investigate the contribution of individual Siglec receptors to viral attachment, we carried out pseudovirus infections in the presence of blocking antibodies against the major macrophage-expressed Siglecs, Siglec-1, -3 and -9. Blocking Siglec-1 or -3 reduced JRFL infection of MDM to 20% and 70%, respectively, compared to the PBS control (Figure 6B). Blocking Siglec-9, however, did not affect the infection, even at an antibody concentration of 100 µg/ml. These data demonstrate a preferential involvement of Siglec-1 in HIV-1 infection of MDM. This is also consistent with the high affinity binding of Siglec-1 to gp120 relative to other Siglecs (Table 1). The striking difference between blocking Siglec-1 and -9 is interesting even though both recombinant proteins bind gp120 well in solution. Their preferential usage by HIV-1 may be related to their differential masking by cell surface cis-sialic acid. This is consistent with our gp120 binding experiment, which shows that the binding of gp120 to Siglec-9 transfected CHO cells requires neuraminidase treatment, unlike Siglec-1 (Figure 3B). Siglec-1 has 17 extracellular domains and is likely less masked than the three-domain Siglec-9. To further address the potential contribution of masked Siglec receptors on macrophages, we treated MDM with neuraminidase prior to HIV-1 infection. Indeed, treating MDM with neuraminidase significantly increased luciferase virus infection for multiple strains of pseudotyped HIV-1 (Figure S4), suggesting a potential contribution by masked Siglecs to the viral infection under certain conditions.

### Viral sialic acids facilitate R5-tropic HIV-1_BaL_ infection of macrophages

To explore if viral sialic acids also facilitate productive HIV-1 infection, we examined the effect of sialyllactose on MDM infection using a replication competent R5-tropic HIV-1_BaL_ virus that was propagated in primary human macrophages. Virus production in human macrophages ensures a physiologically relevant glycosylation compared to that of the 293T cell-produced pseudoviruses. The infections were performed in the presence of sialyllactose, lactose, PBS, or a known entry inhibitor, T20, and evaluated based on viral p24 antigen level in the supernatant collected over a two-week period. Similar to the luciferase virus infections, HIV-1_BaL_ infection of MDM was reduced significantly by sialyllactose compared to PBS or lactose at all time points (Figures 6C–D, Figure S5 & Figure S6).

### Strain variation of R5-tropic viruses affected sialic acid-mediated viral attachment

Compared to the relatively conserved CD4 binding site on gp120, the positions of viral glycan sites are less conserved, implying a potential variation in sialic acid-facilitated viral entry. The heterogeneity of glycosylation modification could further affect the sialic acid content on viral envelopes. The variation in sialic acid-dependent infection is evident when comparing the infection of macrophages by four different strains of R5-tropic HIV-1 (JRFL, DH125, AD8 and HW1) pseudoviruses treated with sodium periodate (Figure 5E). The mild periodate treatment of viruses resulted in a 40–60% reduction in infection by DH125 and AD8, but only a 20% reduction by JRFL and HW1 isolates. In addition, treatment of macrophages with neuraminidase resulted in diverse enhancement of infection among the four strains of viruses (Figure S4), suggesting a strain variation in sialic acid-mediated viral attachment.

### Variation in Siglec expression associated with sialic acid-dependent viral attachment

**Figure 7:**
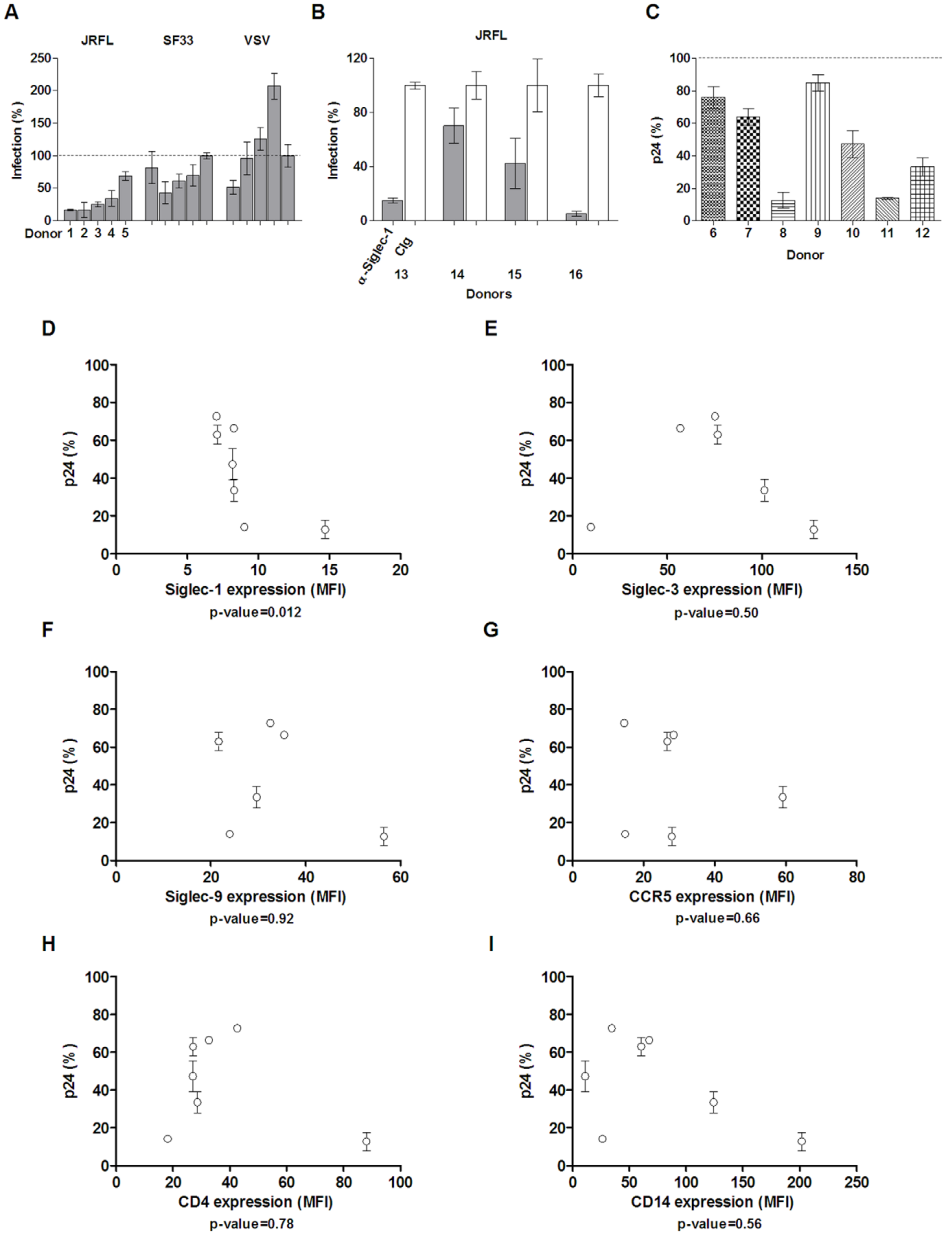
Host variation in pseudovirus and HIV-1_BaL_ infections of MDM. (A) Infections of MDM from five separate donors with JRFL, SF33 or VSV pseudoviruses in the presence of 50 mg/mL SL or PBS. Levels of viral entry (grey bars) are shown as percentage compared to PBS control (shown as a dotted line at 100% level). (B) Infections of MDM from four separate donors with JRFL pseudovirus in the presence of 20–100 µg/mL anti-Siglec-1 antibody (grey bars) or control sheep IgG (white bar). 100 ug/mL α-Siglec-1 was used in experiments with donors 13, 14, and 15; 20 ug/mL α-Siglec-1 was used with donor 16. Results are representative of one out of three experiments. (C) Relative infection levels from seven separate donors compared to their PBS controls on days 12 or 14 post-infection with the HIV-1_BaL_ in the presence of 50 mg/mL SL (patterned bars) or PBS (dotted line at 100% level). (D–I) HIV-1_BaL_ relative infection levels plotted against host expression of Siglec-1 (D), Siglec-3 (E), Siglec-9 (F), CCR5 (G), CD4 (H), and CD14 (I). The error bars on donors 6, 9, and 11 are less than 1% and do not appear in the figure. p-values were generated using Spearman correlation.

In addition to viral strain-associated variation in sialic acid content, variations in host Siglec receptor expression may also affect sialic acid-facilitated viral entry. To address this, we carried out pseudovirus infections (JRFL and VSV) of MDM from five individuals (donors 1–5) in the presence of sialyllactose (Figure 7A). The result showed that the sialyllactose-mediated inhibition of JRFL pseudovirus infections varied between 30%–80% among the five donors. To specifically examine if Siglec-1 contributed to donor variation in sialic-acid dependent entry, similar JRFL pseudovirus infections were carried out in the presence of the anti-Siglec-1 antibody instead of sialyllactose (Figure 7B). The result showed that while the antibody nearly completely blocked the infection of MDM from donor 13, with less than 5% residual infection, the inhibitory effect was more marginal in two of the four donors. Thus, Siglec-1 can play a critical role in HIV-1 infection and may contribute to the observed host variation in sialic acid-dependent viral attachment.

To further examine variations in infection contributed by different hosts, we carried out productive infections of MDM from seven donors (donors 6–12) with HIV-1_BaL_ in the presence of sialyllactose (Figure 7C). Similar to pseudovirus infections, sialyllactose inhibition of HIV-1_BaL_ varied between 10%–80% relative to the control. To delineate the factors important for sialic acid-mediated HIV-1_BaL_ infections, we measured the expression levels of multiple surface markers from these donors and analyzed the statistical association between their expression and the sialyllactose-mediated inhibition of the infections using Spearman correlations (Figures 7D–I). The expression markers, which varied among the seven donors, included the known HIV-1 entry receptors, CD4 and CCR5; the major macrophage sialic acid receptors, Siglec-1, Siglec-3 and Siglec-9; and a lineage-specific marker for macrophages, CD14. If sialyllactose has a specific receptor target, its inhibition effect would be expected to correlate with the expression of the receptor. Conversely, the expression of receptors not associated with sialyllactose would not be expected to correlate with its inhibition outcome. Indeed, the expression of CD14 was not correlated with sialyllactose-mediated inhibition to HIV-1_BaL_ infection with a p-value of 0.56 in a Spearman two-tailed correlation analysis with 95% confidence interval (Figure 7I). Interestingly, the expression of known HIV-1 entry receptors, CD4 and CCR5, also failed to correlate with the inhibition outcome (p-values = 0.78 and 0.66, respectively), suggesting that the sialyllactose-mediated inhibition is not related to the CD4 and CCR5 expression. In contrast, the expression of Siglec-1 showed a direct correlation (p-value = 0.012) with sialyllactose-mediated inhibition of infection (Figure 7D). Higher Siglec-1 expression was associated with increased sialic acid-dependent entry and hence became more sensitive to sialyllactose inhibition. Although the expression correlations of Siglec-3 and -9 failed to reach statistical significance (Figures 7E–F), Siglec-3 expressions appear to generally correlate with the sialyllactose inhibition with the exception of one donor, suggesting a potential contribution of Siglec-3 in certain situations of HIV-1 infection. These results are also consistent with the antibody blocking of JRFL pseudovirus infections showing that Siglec-1 was the major facilitating receptor with possible minor contributions from Siglec-3 to HIV-1 infection (Figure 6B).

## Discussion

Macrophages represent an important target and potentially long-lived reservoir for R5-tropic HIV-1 [3], [4]. Unlike CD4^+^ T cells, which express abundant CD4, macrophages express relatively low levels of the receptor. In this study, we investigated whether HIV-1 utilizes additional attachment receptors to facilitate entry into macrophages. We showed that glycans present on HIV-1 gp120 interact with Siglecs, a family of I-type lectin receptors. Both in solution and on transfected cells, recombinant gp120 proteins recognized Siglecs through gp120-associated sialoglycans independent of CD4 binding. The binding affinities of gp120 for Siglecs varied substantially depending on the isoforms and densities of Siglecs as well as strains of HIV-1. Importantly, the gp120 bindings to Siglec receptors were inhibited by sialyllactose but not lactose. Also, disrupting the sialic acid structure with sodium periodate significantly reduced the bindings. Additional binding experiments using neuraminidase to remove cell surface-associated sialic acid on Siglec-transfected CHO cells showed receptor variations in a phenomenon known as “masking”. Part of the neuraminidase effect is to unmask Siglec receptors on the cell surface thus increasing their binding to viral sialic acids. Siglec-1 transfected CHO cells bound to gp120 without neuraminidase treatment (Figure 3), suggesting that most of Siglec-1 exists in an unmasked state, likely due to its large size with 17 predicted immunoglobulin domains [29]. In contrast, Siglec-9, although a high affinity receptor for sialic acid, displayed significant adhesion only after neuraminidase treatment, suggesting most of the receptors are masked on cell surface.

Consistent with the binding results, HIV-1 R5-pseudovirus infections of MDM were preferentially inhibited by sialo- but not their asialo-compounds and by sodium periodate treatment of the viruses. However, the effects of the sialyllated compounds in X4 infections were less conclusive as X4-viruses infect macrophages at a very low level due to the preferential usage of CXCR4. The results of Siglec blocking antibodies on R5-pseudovirus infection further defined the contribution of individual Siglec receptors in HIV-1 infection of macrophages. The preferential involvement of Siglec-1 and, to a lesser extent, Siglec-3 was observed in JRFL pseudovirus infections blocked by Siglec antibodies, as well as sialyllactose-inhibited HIV-1_BaL_ infections. These results are also consistent with gp120 binding experiments using Siglec-transfected CHO cells. Thus, the contribution of Siglec-1 in HIV-1 infection reflects its high binding affinity to sialic acid and favorable availability for viral attachment.

While the exact distributions of viral sialic acids on HIV-1 isolates remain unknown, their predicted N-glycan sites vary significantly both in positions and numbers. In contrast to conserved CD4 binding site on gp120 [16], half of the glycosylation sites in gp120 variable loop regions are not conserved among different strains (Figure S7). The variations in viral envelope sialyloglycan distribution may very well result in variations in Siglec-dependent viral attachment, consistent with the observed variations in sialic acid-dependent infections among JRFL, DH125, AD8 and HW1 strains of HIV-1. In addition, host factors could influence HIV-1 attachment. Interestingly, the expression of Siglec-1 is up-regulated by inflammation and infection, including HIV-1 infection [24], [37]. This raises the possibility that increased Siglec-1 expression on macrophages at inflammatory sites may further enhance HIV replication and thus contribute to viral dissemination. This is also consistent with a recent finding of an association between immune activation and progressive SIV infection in pigtail macaques [38]. Thus, viral envelope glycan heterogeneities together with host Siglec expressions could lead to a wide range of sialic acid-dependent host susceptibilities to HIV infection.

**Figure 8:**
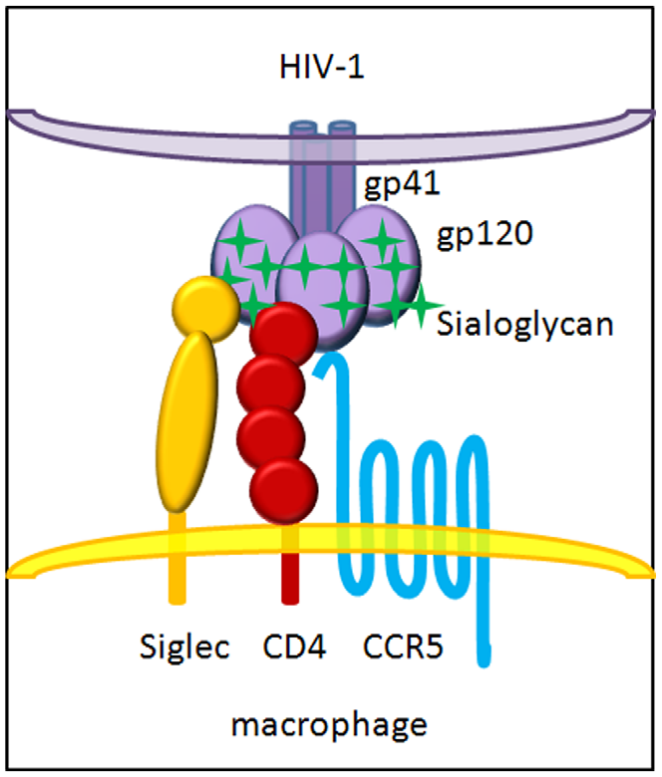
A model displaying the involvement of Siglec receptors in HIV-1 infection of macrophages.

In conclusion, we suggest that HIV-1 viruses utilize their sialoglycans on the envelope protein to facilitate viral attachment to macrophages through binding to Siglec receptors, in particular Siglec-1, and thus enhancing their binding to CD4 for productive entry (Figure 8). While viral glycans are known to shield HIV and SIV from host immune detection as some glycan deletion mutations have resulted in increased sensitivities to neutralizing antibodies [17], [18], our study suggests that HIV-1 viral glycans function not only as a shield to evade host detection but also as a sword to facilitate host entry. This glycan-mediated viral adhesion to host cells resembles physiological cell-cell adhesions between cell surface sialoglycans and the same lectins. Although the number and locations of gp120 sialoglycans important for Siglec recognition remains to be determined, it is tempting to speculate that some of the known entry-deficient glycan mutations in the V1 and V2 loops affect Siglec binding during viral entry. The inhibition of viral-Siglec interactions exhibited by sialic acid-rich compounds shown here suggests a novel class of antiviral compounds based on sialic acid homologs.

## Materials and Methods

### Reagents

Unless otherwise specified, all reagents and chemicals were purchased from Sigma-Aldrich Co. (St. Louis, MO). Recombinant human Siglec-1-Fc was prepared from transiently-transfected COS cells using a plasmid encoding the four N-terminal Ig domains of hSiglec-1 fused to the Fc regions of human IgG_1_ [39]. Blocking antibodies against Siglec-1 and Siglec-9 (sheep polyclonal) were prepared by Rita Sharma in the Crocker laboratory; mouse monoclonal anti-human Siglec-3 (clone 6C5/2) was obtained from Abcam (Cambridge, MA). Other recombinant Siglec-Fc, Siglec-1 (non-Fc fusion), and CD4 proteins were purchased from R&D Systems, Inc. (Minneapolis, MN). The mouse anti-human CD4 monoclonal antibody (Leu3A), antibodies against CD14, CXCR4, CCR5, and their isotype controls (IgG_1_ and IgG_2A_) were obtained from BD Biosciences (San Jose, CA). PE-conjugated mouse anti-human Siglec-1 used for the FACS analysis of MDM was purchased from Santa Cruz Biotechnology (Santa Cruz, CA) or Biolegend (San Diego, CA). FACS antibodies against other Siglecs were obtained from R&D Systems, Inc. or BD Biosciences. Macrophage colony-stimulating factor (M-CSF) and granulocyte-macrophage colony stimulating factor (GM-CSF) were purchased from Peprotech Inc. (Rocky Hill, NJ). Polyacrylamide (PAA)-conjugated model carbohydrates were obtained from Glycotech, Inc. (Rockville, MD). The recombinant gp120 proteins were expressed in CHO cells in monomeric forms as previously described [40], [41]. Recombinant cyanovirin-N was prepared from a bacterial expression plasmid provided by Dr. Mori, as described [42]. Neuraminidase from *Arthrobacter ureafaciens* was purchased from Roche Diagnostics (Indianapolis, IN). Peptide N-glycanase was obtained from Boehringer Mannheim (Amsterdam, Netherlands). CellTiter-Glo Luminescent Cell Viability Assay and Luciferase Assay System were purchased from Promega Corporation (Madison, WI). HIV-1 p24 ELISA kit was obtained from PerkinElmer Life Sciences, Inc. (Waltham, MA). For FACS analysis, recombinant gp120 proteins were labeled with biotin using a biotinylation kit from Pierce Biotechnology (Rockford, IL). RPMI, penicillin/streptomycin, fetal bovine serum, HEPES, and Versene for MDM experiments were purchased from Invitrogen Corporation (Carlsbad, CA).

### Surface plasmon resonance (SPR) measurements

Solution binding studies were performed using a BIAcore 3000 instrument (GE Healthcare). Recombinant Siglec-Fc proteins were immobilized onto carboxymethylated dextran (CM5) surface-based sensor chips by either a capture with immobilized protein A or primary amine-mediated direct surface cross-link. The functional integrity of immobilized Siglec receptors was assessed by their binding to specific antibodies as well as to polyacrylamide (PAA) conjugated model carbohydrates, α(2,3)-sialyllactose (3′SL-PAA), α(2,6)-sialyllactose (6′SL-PAA), and α(2,8)-disialic acid (8′SA-PAA). Recombinant gp120 proteins with varying concentrations between 10–500 nM, in either PBS or HBS-P buffer, were injected over immobilized receptors at a flow rate of 20 µL/min. The dissociation constants were determined from kinetic curve fitting using the BIAevaluation software (GE Healthcare). For competitive binding experiments, various gp120 samples were injected onto captured Siglec sensorchips in the presence of either 1.6 or 16 mM concentrations of α(2,6)-sialyllactose.

### Establishing stable Siglec transfected CHO Cells

CHO-lec3.2.8.1 cells (kindly provided by Dr. Pamela Stanley) [43], were cultured in DMEM/F12 medium supplemented with 5% FBS. pcDNA3.1 vectors containing coding regions for human Siglec-1, -3, -5, -7 and -9, respectively, were transfected into the CHO cells using Lipofectamine 2000 based protocol (Invitrogen Corporation). G418-resistant colonies were cloned, expanded, and screened for cell-surface Siglec expression based on FACS analysis using their respective antibodies. The functionality of the stable Siglec-transfected cells were confirmed by their preferential binding to sheep red blood cells compared to untransfected CHO cells.

### Differentiation of monocyte-derived macrophages (MDM)

Elutriated human monocytes were obtained from randomly selected non-identified healthy donors from the Department of Transfusion Medicine, National Institutes of Health. The monocytes were plated at 1×10^7^ in 10 cm dishes in 10 mL RPMI without serum. After attachment at 37°C for one hour, differentiation was initiated in RPMI containing 10% FBS, 1% penicillin/streptomycin with 1 ng/mL M-CSF and GM-CSF [44]. Culture media was replaced on days 4 or 5 and on the day prior to infection (usually day 7). Differentiated MDM were detached from the plates using Versene and cell scrapers, and then washed twice with RPMI. Total cell counts and viability determinations were assessed with the Guava Personal Cell Analysis System (Guava Technologies); all assays were performed with a cellular viability greater than 70%. The differentiated MDM consistently displayed the surface expression of CD33^high^, CD14^+^, CD4^+^, CCR5^+^, and CXCR4^low^ by FACS analysis. CCR5 expression on MDM was increased in the presence of GM-CSF [45]. Siglec receptor expression was evaluated on monocytes and MDM using PE-conjugated mouse anti-human Siglec-1, -3, -5, -7, and -9 with PE-conjugated mouse IgG_1_ or IgG_2A_ as their isotype controls. All samples were blocked with murine IgG prior to triple-staining with FITC-conjugated mouse anti-human CD14 and APC-conjugated mouse anti-human CD4.

### Cell-surface binding analysis

All incubations were carried out on ice. The staining and wash buffer was PBS containing 0.25% BSA, 1 mM EDTA and 0.1% NaN_3_. Analyses were performed using a Becton-Dickinson FACS Calibur flow cytometer (BD Biosciences). CHO wild-type cells, Siglec-expressing CHO cells, and Versene-lifted MDMs were untreated or treated with 0.4 U/mL of neuraminidase for 2 hours at 37°C. After washing, CHO cells were blocked with 0.2 mg/mL murine IgG; MDMs were blocked with 0.2 mg/mL human IgG in the presence or absence of a CD4 blocking antibody (5 µg/mL). Cells were then incubated for 30 minutes with biotin-labeled gp120 followed by PE-conjugated streptavidin. For blocking tests, cells were incubated with blocking reagents prior to the incubation with gp120. To remove gp120-associated sialic acids, proteins were treated with neuraminidase (0.4 U/mL, 2 hours at 37°C) or periodate (1 mM, in dark at room temperature for 10 minutes and terminated by glycerol) before incubation with cells.

### Preparation of the pseudotyped HIV viruses

Single-round replication-competent luciferase viruses pseudotyped with various HIV-1 and VSV envelopes were generated. The HIV viral vector, pNL4-3.Luc.R-E-, which contains a firefly luciferase gene inserted into the NL4-3 HIV nef gene and frameshift mutations to render it as env^−^, was used to generate all pseudotyped viruses [46], [47]. In brief, the expression vectors for pNL4-3.Luc.R-E-, the amphotropic envelope pHEF-VSVG, and the R5-tropic HIV JRFL envelope were obtained through the NIH AIDS Research and Reference Reagent Program. Expression vectors for other HIV-1 envelopes, including the dual-tropic isolate DH125 and R5-tropic isolate AD8, were obtained from M. Martin [48]. The R5-tropic isolate HW1 and its related X4-tropic HW2 counterpart were generated on the backbone of the X4-tropic SF33 expression vector, pTEJ8-SD-envSF33, as previously described [33]. Recombinant HIV luciferase viruses were generated by co-transfecting 293T cells with 5 µg of the NL4-3 backbone and either 5 µg of the HIV envelopes or 1.5 µg of the VSV envelope, as previously described [33]. Virus collected in the culture supernatant were quantified by HIV p24 ELISA and adjusted to 1 µg/mL p24.

### Single-round infection assay

MDM were resuspended at 2×10^6^/mL in culture media. Aliquots of 200 µL (4×10^5^ cells) were transferred to 15 mL tubes for incubation with sialyllactose (Sigma-Aldrich A0828), lactose, cyanovirin-N, fetuin, asialofetuin, and EDTA, individually, at room temperature for 15 minutes prior to the addition of virus. Luciferase viruses pseudotyped with envelopes from R5- and X4-tropic HIV-1 and VSV were added to the cells at a concentration of 100 ng/mL HIV p24. Where appropriate, viruses were individually incubated with recombinant Siglec proteins, mild periodate oxidation, or glycerol, prior to incubation with cells. After incubating cells and virus at 37°C for three hours with occasional agitation, the cells were washed twice with a wash buffer (PBS, 2% FBS, 1% penicillin/streptomycin, 12 mM HEPES), and once with culture media. The infected MDM were then distributed into 96-well plates in triplicate, incubated for 72 hours, lysed, and assayed for luciferase activity according to manufacturer's recommendations (Promega). The concentrations of compounds used in pseudovirus infections are as follows: 1–50 mg/mL of sialyllactose, 50 ng/mL recombinant cyanovirin-N, 1 mg/mL fetuin, 1 mg/mL asialofetuin, 100 µg/mL of the recombinant Siglec-3, -7 and -9, 1 mM NaIO_4_, 10 mM glycerol and 2 mM EDTA. For the infections, MDM were not treated with neuraminidase unless specified. In that case, 0.5 U/mL of the enzyme was incubated with MDM for up to 2 hours at 37°C, and then removed by centrifugation, prior to the addition of virus. The mild periodate oxidation was carried out with the addition of NaIO_4_ to the virus for 10 minutes and followed by neutralization of the periodate with glycerol [31]. For the Siglec blocking antibody experiments, purified human IgG at 2 ug/mL was used to block non-specific binding for 15 min prior to the addition of individual blocking antibodies or controls. 20, 50, or 100 µg/mL of Siglec-1, -3, and -9 blocking antibodies, or 100 µg/mL of mouse or sheep IgG were used. The toxicity of each inhibitory compound was assayed under conditions identical to the infection assay by replacing virus with PBS. Cell viability was determined with Cell Titer-Glo Luminescent Cell Viability Assay according to the manufacturer's recommendations (Promega). No measurable toxicities were detected for sialyllactose at 100 mg/mL, neuraminidase at 1 U/mL, cyanovirin-N at 50 ng/mL, fetuin at 1 mg/mL, asialofetuin at 1 mg/mL, and recombinant Siglec-3, -7 and -9 each at 100 µg/mL concentrations. The concentrations that resulted in less than 20% reduction in cellular viability were determined for periodate, EDTA, and glycerol to be 2 mM, 5 mM, and 20%, respectively.

### HIV-1_BaL_ infection of macrophages

The R5-tropic Ba-L strain of HIV-1 virus (HIV-1_BaL_), propagated using primary human macrophages, was purchased from Advanced Biotechnologies Inc. (Columbia, MD). MDM were resuspended at 3×10^6^/mL in culture media. Cells were incubated at room temperature for 15 minutes with a test compound or appropriate control. The concentrations of compound or control used were: 50 mg/mL of sialyllactose or lactose, 100 ug/mL T20 (kindly provided by Dr. Tae-Wook Chun), or PBS. Cells were exposed to HIV-1_BaL_, at concentrations of 500 (1X), 125 (1/4X), or 32.5 (1/8X) TCID_50_ (median tissue culture infective dose) per 10^6^ cells at 37°C for 1 hour, followed by extensive washing. HIV-1_BaL_ was shown to infect in a dose-dependent manner based on concentrations used (Figures S6). The MDM were then cultured in triplicate in 96-well plates (250 uL/well) and incubated for 14 days. Culture supernatants were collected (100 uL) and wells were replenished with fresh media on days 1, 3, 6, 9, 12, and 14 post-infection (DPI). HIV-1 p24 activity was assayed via ELISA according manufacturer recommendations (Perkin Elmer). All statistical analyses were carried out using the software Prism 5.05 (GraphPad Software, Inc.).

## Supporting Information

Figure S1 Expression of Siglecs on transfected CHO cells. The expression of Siglecs on transfected (grey shaded) and untransfected (open histograms) CHO cells, stained with their respective antibodies.
(DOC)

Figure S2 BIAcore binding between Siglec-3 and gp120. (A) Binding of recombinant gp120 from 92US715 (0.65 µM), 92UG21-9 (0.49 µM), and PBj1.9 (0.2 µM) HIV-1 isolates onto protein A-captured recombinant Siglec-3 Fc fusion protein. The sensorgrams in each panel represent Siglec-3 captured in flow cells 2, 3, and 4 at various levels. Flow cell 1 has immobilized protein A but no captured receptor (blank). (B) Binding of serial dilutions of 92US715 gp120 between 0.43–0.054 µM or 8′SA-PAA between 0.062–20 µM onto immobilized Siglec-3 under high immobilization density. All sensorgrams are shown in response units (vertical axis) versus sample injection time (horizontal axis) in seconds. All dissociation constants are listed in Table 1.
(DOC)

Figure S3 Effect of recombinant Siglec proteins and cyanovirin-N (CVN) on R5- and X4-tropic HIV-1 and VSV. Single-round infection of MDM with JRFL, SF33 and VSV pseudoviruses in the presence of 100 µg/mL recombinant Siglec-3, Siglec-7, Siglec-9, CVN (50 ng/mL), or PBS (100%).
(DOC)

Figure S4 The entry of various R5-(left side) and X4-(right side) tropic HIV and VSV pseudoviruses into MDM with (grey) or without (white) prior treatment with 0.5 U/mL neuraminidase for 1 hour. The observed enhancement in infections upon neuraminidase treatment of MDM is consistent with previously published findings [22], [23]. While the exact mechanism of neuraminidase-mediated enhancement in infection remains unresolved, much of the effect was attributed to the reduction in charge repulsion between viral and host sialic acids [22], [49]. However, our binding results suggest that part of the neuraminidase effect is to unmask cell surface Siglec receptors thus increasing their binding to viral sialic acids. Indeed, cell surface-associated sialidase expression could be induced during monocyte to macrophage differentiation [50].
(DOC)

Figure S5 Effect of sialyllactose (SL) compared to lactose on HIV-1_BaL_ infection of MDM. Infection of MDM with HIV-1_BaL_ (125 TCID_50_) in the presence of 50 mg/mL sialyllactose (light grey squares) or lactose (black circles), or 100 µg/mL T20 (dark grey triangles). The results are shown as the level of HIV-1 p24 (ng/mL) sampled over 14 days post infection (DPI).
(DOC)

Figure S6 Dose-dependent effect of sialyllactose (SL) on HIV-1_BaL_ infection of MDM. Infection of MDM with 500 TCID_50_ (black circles), 125 TCID_50_ (light grey squares), and 31.25 TCID_50_ (dark grey triangles) HIV-1_BaL_ in the presence of 50 mg/ml sialyllactose (A) or PBS (B) over 14 days. The results are shown as the level of HIV-1 p24 (ng/mL) sampled over 14 days post infection (DPI).
(DOC)

Figure S7 Amino acid sequences of R5 HIV-1 envelope gp120 from AD8, JRFL and DH125 strains. Predicted N-glycan sites are highlighted in red. Regions corresponding to variable loops V1–V5 are indicated. Significant variations in predicted N-glycan sites exist among the sequences, particularly within the V1 and V4 loops.
(DOC)
