# Supplementary figures and images for: Siglecs Facilitate HIV-1 Infection of Macrophages through Adhesion with Viral Sialic Acids
Source: PLoS One. 2011 Sep 8;6(9):e24559. doi: 10.1371/journal.pone.0024559 (PMC3169630; doi:10.1371/journal.pone.0024559)

Siglec-1 Siglec-3 Siglec-5 Siglec-7 Siglec-9

Log fluorescence intensity


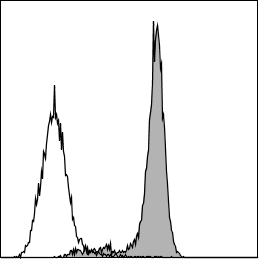

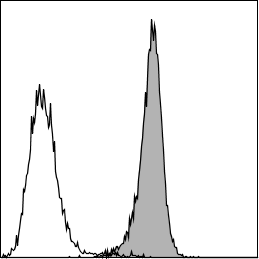

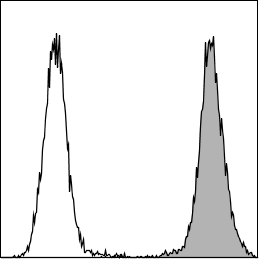


Cell number

250

200

150

100

50

0

100 101 102 103


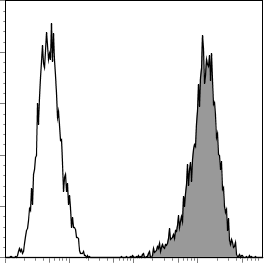


Figure S1

Figure S1

Supplement: Figure S1 — Expression of Siglecs on transfected CHO cells. The expression of Siglecs on transfected (grey shaded) and untransfected (open histograms) CHO cells, stained with their respective antibodies. (DOC) [file pone.0024559.s001.doc]
