# Supplementary material for: Siglecs Facilitate HIV-1 Infection of Macrophages through Adhesion with Viral Sialic Acids
Source: PLoS One. 2011 Sep 8;6(9):e24559. doi: 10.1371/journal.pone.0024559 (PMC3169630; doi:10.1371/journal.pone.0024559)
Supplement: Figure S2 — BIAcore binding between Siglec-3 and gp120. (A) Binding of recombinant gp120 from 92US715 (0.65 µM), 92UG21-9 (0.49 µM), and PBj1.9 (0.2 µM) HIV-1 isolates onto protein A-captured recombinant Siglec-3 Fc fusion protein. The sensorgrams in each panel represent Siglec-3 captured in flow cells 2, 3, and 4 at various levels. Flow cell 1 has immobilized protein A but no captured receptor (blank). (B) Binding of serial dilutions of 92US715 gp120 between 0.43–0.054 µM or 8′SA-PAA between 0.062–20 µM onto immobilized Siglec-3 under high immobilization density. All sensorgrams are shown in response units (vertical axis) versus sample injection time (horizontal axis) in seconds. All dissociation constants are listed in Table 1. (DOC) [file pone.0024559.s002.doc]

A

Moderate Immobilization Levels

High Immobilization Levels

92US715

92UG21-9

PBj1.9

92US715

8’SA-PAA

B

C

.

Figure S2
