# Supplementary material for: Siglecs Facilitate HIV-1 Infection of Macrophages through Adhesion with Viral Sialic Acids
Source: PLoS One. 2011 Sep 8;6(9):e24559. doi: 10.1371/journal.pone.0024559 (PMC3169630; doi:10.1371/journal.pone.0024559)
Supplement: Figure S4 — The entry of various R5-(left side) and X4-(right side) tropic HIV and VSV pseudoviruses into MDM with (grey) or without (white) prior treatment with 0.5 U/mL neuraminidase for 1 hour. The observed enhancement in infections upon neuraminidase treatment of MDM is consistent with previously published findings [22], [23]. While the exact mechanism of neuraminidase-mediated enhancement in infection remains unresolved, much of the effect was attributed to the reduction in charge repulsion between viral and host sialic acids [22], [49]. However, our binding results suggest that part of the neuraminidase effect is to unmask cell surface Siglec receptors thus increasing their binding to viral sialic acids. Indeed, cell surface-associated sialidase expression could be induced during monocyte to macrophage differentiation [50]. (DOC) [file pone.0024559.s004.doc]

Figure S4
