# Supplementary material for: Siglecs Facilitate HIV-1 Infection of Macrophages through Adhesion with Viral Sialic Acids
Source: PLoS One. 2011 Sep 8;6(9):e24559. doi: 10.1371/journal.pone.0024559 (PMC3169630; doi:10.1371/journal.pone.0024559)
Supplement: Figure S5 — Effect of sialyllactose (SL) compared to lactose on HIV-1BaL infection of MDM. Infection of MDM with HIV-1BaL (125 TCID50) in the presence of 50 mg/mL sialyllactose (light grey squares) or lactose (black circles), or 100 µg/mL T20 (dark grey triangles). The results are shown as the level of HIV-1 p24 (ng/mL) sampled over 14 days post infection (DPI). (DOC) [file pone.0024559.s005.doc]

Figure S5

Figure S5
