# Supplementary material for: Siglecs Facilitate HIV-1 Infection of Macrophages through Adhesion with Viral Sialic Acids
Source: PLoS One. 2011 Sep 8;6(9):e24559. doi: 10.1371/journal.pone.0024559 (PMC3169630; doi:10.1371/journal.pone.0024559)
Supplement: Figure S6 — Dose-dependent effect of sialyllactose (SL) on HIV-1BaL infection of MDM. Infection of MDM with 500 TCID50 (black circles), 125 TCID50 (light grey squares), and 31.25 TCID50 (dark grey triangles) HIV-1BaL in the presence of 50 mg/ml sialyllactose (A) or PBS (B) over 14 days. The results are shown as the level of HIV-1 p24 (ng/mL) sampled over 14 days post infection (DPI). (DOC) [file pone.0024559.s006.doc]

A

B

Figure S6
