# Supplementary material for: Siglecs Facilitate HIV-1 Infection of Macrophages through Adhesion with Viral Sialic Acids
Source: PLoS One. 2011 Sep 8;6(9):e24559. doi: 10.1371/journal.pone.0024559 (PMC3169630; doi:10.1371/journal.pone.0024559)
Supplement: Figure S7 — Amino acid sequences of R5 HIV-1 envelope gp120 from AD8, JRFL and DH125 strains. Predicted N-glycan sites are highlighted in red. Regions corresponding to variable loops V1–V5 are indicated. Significant variations in predicted N-glycan sites exist among the sequences, particularly within the V1 and V4 loops. (DOC) [file pone.0024559.s007.doc]

AD8 MRVK---EKYQHLWRWGWKWGTMLLGILMICSATEKLWVTVYYGVPVWKEATTTLFCASD 57

JRFL MRVKGIRKNYQHLWRG----GTLLLGIIVICSAVEKLWVTVYYGVPVWKEATTTLFCASD 56

DH12_5 MRVMGIRKNYQHLWKG----GTLLLGILMICSAAEQLWVTVYYGVPVWKEA**N**TTLFCASD 56

HW1 MRARETRKNYQCLWRW----GTMLLGMLMICSAAENLWVTVYYGVPVWKEATTTLFCASD 56

AD8 AKAYDTEVHNVWATHACVPTDPNPQEVVLE**N**VTENFNMWKNNMVEQMHEDIISLWDQSLK 117

JRFL AKAYDTEVHNVWATHACVPTDPNPQEVVLG**N**VTEKFNMWKNNMVEQMQEDIISLWDQSLK 116

DH125 AKAYDTEVHNVWATHACVPTDPNPQEILLE**N**VTEDFNMWKNNMVEQMHEDIISLWDQSLK 116

HW1 AKAYKTEVHNVWATHACVPTDPNPQEIELE**N**VTENFNMWKNDMVEQMQEDIISLWDQSLK 116

V1 V2

AD8 PCVKLTPLCVTL**N**CTDLR**N**VTNI**NN**SSE-------GMRGEIK**N**CSFKITTSIRDKVKKDY 173

JRFL PCVKLTPLCVTLNCKDV**N**AT**N**TT**N**GSEG------TMERGEIK**N**CSF**N**ITTSIRDEVQKEY 173

DH125 PCVKLTPLCVTLHCTDLK**N**GTNLK**N**GTK---IIGKSMRGEIK**N**CSF**N**VTKNIIDKVKKEY 176

HW1 PCVELTPLCVTLNCIDV**N**ITNT**N**SSN**NN**TTRKMDKEMIGELK**N**CSF**N**ITTNIRDKVQKEF 176

AD8 ALFYRLDIVPINND**N**TSYRLINC**N**TSTITQACPKVSFEPIPIHYCTPAGFAILKCKDKKF 233

JRFL ALFYKLDVVPIDN**NN**TSYRLISCDTSVITQACPKISFEPIPIHYCAPAGFAILKCNDKTF 233

DH125 ALFYRHDVVPIDR**N**ITSYRLISC**N**TSTLTQACPKVSFEPIPIHYCAPAGFAILKCKDKKF 236

HW1 ALFYKLDVVPI**N**NSNANYRLISC**N**TSVIKQACPKVSFEPIPIHYCAPAGFAILQCNDKKF 236

AD8 **N**GTGPCK**N**VSTVQCTHGIRPVVSTQLLL**N**GSLAEEEVVIRSS**N**FTDNAKNIIVQLKESVE 293

JRFL NGKGPCK**N**VSTVQCTHGIRPVVSTQLLL**N**GSLAEEEVVIRSD**N**FTNNAKTIIVQLKESVE 293

DH125 **N**GTGPCT**N**VSTVQCTHGIRPVVSTQLLL**N**GSLAEEEVVIRSS**N**FTDNAKIIIVQL**N**ETVE 296

HW1 NGKGPCT**N**VSTVQCTHGIRPVVSTQLLL**N**GSLAEEEVVIRSD**N**ITDNAKNIIVQLKESVE 296

V3

AD8 I**N**CTRPN**N**NTRKSIHIGPRRAFYTTGKIIGDIRQAHC**N**ISRTKW**N**NTLNQIATKLKEQFGN 351

JRFL I**N**CTRPN**N**NTRKSIHIGPGRAFYTTGEIIGDIRQAHC**N**ISRAKW**N**DTLKQIVIKLREQFE 351

DH125 I**N**CTRPN**N**NTRKGITLGPGRVFYTTGEIVGDIRKAHC**N**ISKVKWHNTLKRVVEKLREKFE 354

HW1 I**N**CTRPN**N**NTRRGIHIGPGSAFYATGDIIGDIRQAHCTI**N**GTEW**N**NTLEQIVEKLREQFK 356

V4

AD8 **N**KTIVF**N**QSSGGDPEIVMHSFNCGGEFFYC**N**STQLF**N**STWNF**N**GTW**N**LTQS**N**GTEG**N**DTI 411

JRFL **N**KTIVF**N**HSSGGDPEIVMHSFNCGGEFFYC**N**STQLF**N**STW----N**N**NTEGS**N**NTEGN-TI 405

DH125 **N**KTIVF**N**KSSGGDPEIVMHSFNCGGEFFYCNTKKLF**N**STW-----**N**GTEGSYNIEG**N**DTI 408

HW1 **N**KTIVFTHSSGGDPEIVMHSFNCGGEFFYC**N**TTKLF**N**STWWS-----**N**NTWKGTEMLE**NL** 410

V5

AD8 TLPCRIKQIINMWQEVGKAMYAPPIRGQIRCSS**N**ITGLILTRDGGT**N**SSG-SEIFRPGGG 471

JRFL TLPCRIKQIINMWQEVGKAMYAPPIRGQIRCSS**N**ITGLLLTRDGGINE**N**GT-EIFRPGGG 465

DH125 TLPCRIKQIINMWQEVGKAMYAPPISGQIWCSS**N**ITGLLLTRDGGK**N**SS--TEIFRPGGG 467

HW1 TLPCRIKQIIRMWQRVGKAMYAPPIRGRISCLS**N**ITGLLLTRDGGSN**N**GSDTEIFRPGGG 470

AD8 DMRDNWRSELYKYKVVKIEPLGVAPTKAKRRVVQREKRAVGTIGAMFLGFLGAAGSTMGAA 531

JRFL DMKDNWRSELYKYKVVKIEPLGVAPTKAKRRVVQREKRAVG-IGAVFLGFLGAAGSTMGAA 524

DH125 DMRDNWRSELYKYKVVRVEPLGIAPTKAKRRVVQREKRAVG-IGAVFLGFLGAAGSTMGAA 526

HW1 DMRDNWRSELYKYKVVKIEPLGVAPTKAKRRVVQREKRAVGVIGAMFLGFLGAAGSTMG 530

Figure S7

Figure S7
